# Establishing a small animal model for evaluating protective immunity against mumps virus

**DOI:** 10.1371/journal.pone.0174444

**Published:** 2017-03-31

**Authors:** Adrian Pickar, Pei Xu, Andrew Elson, James Zengel, Christian Sauder, Steve Rubin, Biao He

**Affiliations:** 1 Department of Infectious Diseases, College of Veterinary Medicine, University of Georgia, Athens, Georgia, United States of America; 2 Center for Biologics Evaluation and Research, Food and Drug Administration, Silver Spring, Maryland, United States of America; University of Iowa, UNITED STATES

## Abstract

Although mumps vaccines have been used for several decades, protective immune correlates have not been defined. Recently, mumps outbreaks have occurred in vaccinated populations. To better understand the causes of the outbreaks and to develop means to control outbreaks in mumps vaccine immunized populations, defining protective immune correlates will be critical. Unfortunately, no small animal model for assessing mumps immunity exists. In this study, we evaluated use of type I interferon (IFN) alpha/beta receptor knockout mice (IFN-α/βR^−/−^) for such a model. We found these mice to be susceptible to mumps virus administered intranasally and intracranially. Passive transfer of purified IgG from immunized mice protected naïve mice from mumps virus infection, confirming the role of antibody in protection and demonstrating the potential for this model to evaluate mumps immunity.

## Introduction

Mumps virus (MuV) is a member of the *Paramyxoviridae* family, Rubulavirus genus. MuV has a nonsegmented, negative-stranded RNA genome of 15,384 nucleotides that encodes 9 viral proteins [1]. MuV is a contagious human pathogen. The hallmark for MuV infection is parotitis, however about one third of infections occur without recognized symptoms [2, 3]. MuV is also highly neurotropic where invasion of the central nervous system (CNS) has been demonstrated to occur in more than half of all clinical cases in some studies. Despite the apparent frequency of CNS infections, clinical manifestations are relatively uncommon, with asceptic meningitis occurring in approximately 10% of cases and encephalitis in less than 0.5% [1]. There is no specific antiviral therapy for mumps infection and treatment is generally provided to alleviate disease symptoms.

At least 12 strains of MuV are administered as live attenuated vaccines throughout the world [4]. Mumps vaccines are typically given in combination with other vaccines, such as the trivalent measles, mumps, and rubella vaccine (MMR). The Jeryl Lynn vaccine strain (JL) is the most widely used globally and the only strain ever used in the United States (licensed in 1967) [1]. Even though the JL vaccine has been shown to be safe and efficacious [1, 5–9], recent large outbreaks have occurred in the United States and worldwide despite high vaccine coverage [10–13]. After nearly a decade of only a few hundred mumps cases being reported annually in the US, in 2006 over 6,500 mumps cases were reported [14]. The overwhelming majority of these cases had a history of previous mumps vaccination. Similarly, a mumps outbreak occurred in New York and New Jersey in 2009–2010 where 88% of the patients had previously received at least one-dose of mumps vaccine and 75% of patients had two doses [15]. This has raised concerns as to the efficacy of the current vaccine program in the United States, a problem made difficult to investigate due to the absence of an established serologic correlate of protection, such as a specific level of mumps neutralizing antibody predictive of protection. Not only does the lack of immune correlates of protection make it difficult to investigate factors contributing to such outbreaks, but also impedes development of new, potentially more effective vaccines.

Currently, the only animal model for a natural, systemic infection of MuV and in which mumps immunity could potentially be studied is the rhesus macaque [16]. This highlights the importance of developing and validating a small animal model in which such studies can more readily and more humanely be carried out.

Based on previous work by others, mumps viruses do not productively replicate in mice nor cause any symptomatology [17, 18]. Similar problems with other RNA viruses, such as Junin virus, Dengue, and Ebola has been overcome by using mice lacking the IFN-α/β receptor (IFN-α/βR^−/−^) [19–21]. Given that MuV replication is known to be highly susceptible to the antiviral action of alpha/beta interferons (IFN-α/β) [22–25], we sought to investigate use of IFN-α/βR^−/−^ mice to study mumps virus. In this study, we have examined infection routes of IFN-α/βR^−/−^ mice with a clinical isolate from a 2006 MuV outbreak (MuV^Iowa/US/06^, referred to here as MuV-IA). In addition, we have examined the protective efficacy of neutralizing antibody to MuV-IA in reducing lung viral load.

## Materials and methods

### Viruses and cells

Vero cells were maintained in Dulbecco’s modified Eagle medium (DMEM) with 5% fetal bovine serum (FBS) and 1% penicillin-streptomycin (P/S) (Mediatech Inc., Manassas, VA). Cells were incubated at 37°C with 5% CO_2_ and passed at an appropriate dilution 1 day prior to use to achieve 80% to 90% confluence the next day. The MuV strain, MuV^Iowa/US/06^ (referred to here as MuV-IA), and a recombinant version of this virus expressing a Renilla luciferase protein (rMuV-RLuc) were described and grown in Vero cells as before [26]. Virus was harvested 4 days post infection (dpi) and supplemented with 1% BSA, then stored at -80°C. Viral titers were measured in Vero cells by plaque assay.

### rMuV-RLuc infection of mice

All mouse experiments were performed according to the protocols approved by the Institutional Animal Care and Use Committee at the University of Georgia. IFN-α/βR^−/−^ mice (B6.129S2-Ifnar1tm1Agt/Mmjax) on the C57BL/6 background were purchased from The Jackson Laboratory (Bar Harbor, ME, USA), bred in the animal facility at the University of Georgia, and used for all challenge experiments. Control C57BL/6 mice and BALB/c mice were purchased from Harlan Laboratories (Indianapolis, IN) and used for challenge experiments, where indicated.

IFN-α/βR^−/−^ mice were infected with rMuV-RLuc by the subcutaneous route (s.c.) with 6x10^6^ PFU in 200μL, intraperitoneal route (i.p.) with 3x10^6^ PFU in 100μL, intranasal route (i.n.) with 3x10^6^ PFU in 100μL, intracranial route (i.c.) with 1000 PFU in 20μL, or mock infected with PBS in the respective volume for each route. Lungs, spleens, and brains were harvested from mice 4, 7, and 10 dpi (s.c., i.p., and i.n. routes) or 2, 4, and 6 dpi (i.c. route) and added to 1 mL of DMEM supplemented with 1% bovine serum albumin (BSA) in gentleMACS M tubes (Miltenyi Biotec Inc., Auburn, CA) and stored on ice. Tissues were homogenized using the Protein.01 program of a gentleMACS Dissociator (Miltenyi Biotec Inc.), centrifuged at 3000 × g for 10 minutes and stored at -80°C. Viral titers were measured in Vero cells by plaque assay.

### Renilla luciferase assay

Tissue homogenates were centrifuged at 12,000 rpm for 5 minutes. 40 μL of cleared homogenates was used to carry out the Renilla luciferase assay with 50 μL Renilla luciferase assay reagent (Promega, Madison, WI) and light intensity was detected by a GloMax® 96 Microplate Luminometer (Promega). Four replicates of each tissue sample from each mouse were averaged. Due to differences in homogenates, background levels of luciferase varied.

### Intranasal infection for kinetics study

C57BL/6 control and IFN-α/βR^−/−^ mice (males and females, 6-to-8 weeks old) were challenged i.n. with 1x10^6^ PFU of MuV-IA in 100 μL. Lungs were harvested from mice 2, 3, and 4 dpi and added to 1 mL of DMEM supplemented with 1% BSA. Tissues were homogenized and processed as described above. Individual tubes were weighed before and after addition of tissues to calculate lung mass.

### Preparation of serum from immunized mice

BALB/c mice (females, 6-to-8 weeks old) were immunized i.n. with 1x10^6^ PFU of MuV-IA in a volume of 100 μL or mock immunized with 100 μL PBS. Mice were boosted at 3 and 6 weeks post immunization (wpi), then bled and euthanized at 8 wpi. Serum was pooled, heated at 56°C for 30 minutes, and stored at -20°C.

### Preparation of IgG from immunized mice

BALB/c mice (females, 6 to 8 weeks old) were immunized i.n. with 1x10^6^ PFU of MuV-IA in a volume of 100 μL or mock immunized with 100 μL PBS. Mice were boosted at 3 and 5 wpi, then bled and euthanized at 8 wpi. Serum was pooled and IgG was purified using NAb protein A/G spin columns according to manufacturer’s protocol (Thermo Scientific, Rockford, IL). Column eluate was collected during IgG purification (referred to as serum eluate). Purified IgG was dialyzed in PBS using a Slide-A-Lyzer dialysis cassette (Thermo Scientific).

### Passive transfer experiment

IFN-α/βR^−/−^ mice (males and females, 6-to-8 weeks old) received passive transfer by the i.p. route with 300 μL of pooled serum from MuV-IA immunized mice (50 μL and 150 μL groups received serum diluted in PBS for 300 μL total volume), 300 μL pooled serum from PBS immunized mice, or 1 mL of PBS, serum eluate or purified IgG. Mice were challenged 24 hours post passive transfer i.n. with 1x10^6^ PFU of MuV-IA in a volume of 100 μL. Lungs and serum were collected from mice 2 dpi and processed as described above.

### Plaque assay

Viral titers were measured in lung homogenates by plaque assay. Lung homogenates were spun at 12,000 rpm for 5 min and 100 μL of undiluted or diluted (1:10) cleared homogenates were used to inoculate Vero cells in triplicate. Cells were incubated at 37°C with 5% CO_2_ for 1–2 hours (h). The inoculating mixture was removed and replaced with 4 ml DMEM containing 2% FBS, 1% P/S, and 1% low-melting-point agarose. At 7 dpi, the plaques were fixed in 1% formaldehyde in PBS, stained with crystal violet, and counted.

### Plaque reduction neutralization test

Serum samples were heated at 56°C for 30 minutes and serially diluted 2-fold from 1:2 to 1:4096 in DMEM plus 1% BSA. MuV-IA was diluted to 4,000 PFU/mL in DMEM plus 1% BSA. One portion of serum (80 μL) was mixed with an equal volume of MuV-IA containing 320 PFU of virus and placed into each well of a 96-well plate and incubated at 37°C with 5% CO_2_. After 1 h of incubation, 50 μL of the incubation mix or diluted virus (back titration) was added into 6-well plates of Vero cells for plaque assay infection as described above. Two replicates for each dilution were used.

### ELISA

Enzyme-linked immunosorbent assay (ELISA) was performed using Immulon 2HB 96-well microtiter plates (ThermoLab Systems) coated with MuV-IA proteins at 2 μg/ml or serial dilutions of unlabeled mouse IgG, IgA, or IgM standards (SouthernBiotech, Birmingham, Al) and incubated at 4°C overnight. Plates were washed with 1% Tween in PBS (PBST) and each well was blocked with 200 μl PBST with 3% nonfat dry milk (Blotto) for 1 h at room temperature (RT). Serum samples were inactivated by heating at 56°C for 30 min and were serially diluted 2-fold in Blotto. 100 μl of diluted serum samples were transferred to the MuV-IA coated plates and incubated for 2 h at RT. To quantify anti-MuV-IA specific antibodies, horseradish peroxidase (HRP)-labeled, goat anti-mouse IgG (1:2000 dilution), IgA (1:500), or IgM (1:500) (SouthernBiotech) was diluted in Blotto and 100 μl was added to each well. Following incubation for 1 h at RT, plates were washed and developed by adding 100 μl SureBlue Reserve TMB Microwell Peroxidase Substrate (KPL Inc., Gaithersburg, MD) per well. The OD was measured at 405 nm or 450 nm using a BioTek Epoch microplate reader. Quantification of serum IgG, IgA, and IgM levels was calculated based on the linear range of the respective antibody standards.

### Statistical analysis

Statistical analysis was performed using GraphPad Prism version 6.00 for Windows (GraphPad Software, San Diego, CA). ANOVA and Tukey multiple comparison tests were used to calculate *P* values.

## Results

### IFN-α/βR^−/−^ mice susceptible to rMuV-RLuc by intranasal and intracranial infection routes

**Fig 1:**
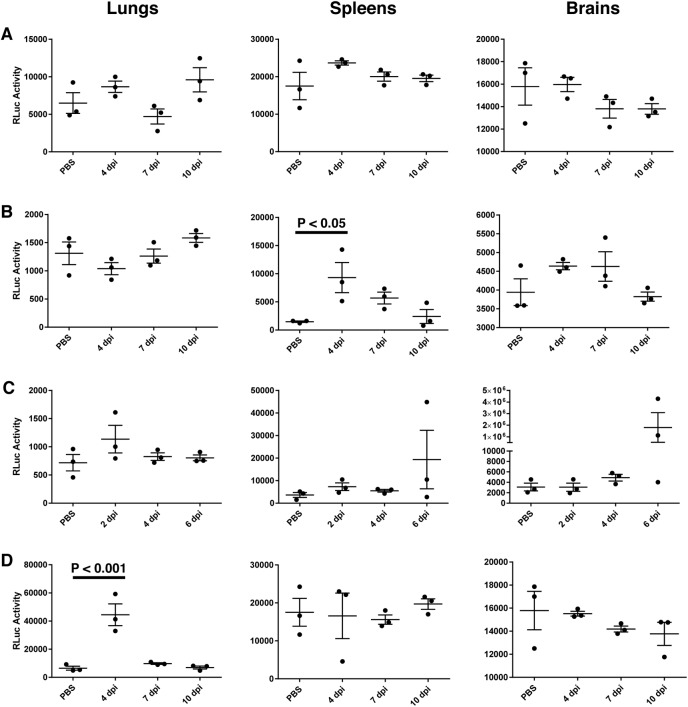
Susceptibility of IFN-α/βR^−/−^ mice to rMuV-RLuc by various inoculation routes. IFN-α/βR^−/−^ mice were infected with (A) 6x10^6^ PFU rMuV-RLuc in 200μL s.c., (B) 6x10^6^ PFU in 200μL i.p., (C) 1000 PFU in 20μL i.c., (D) 3x10^6^ PFU in 100μL i.n., or mock infected with PBS in the respective volume for each route. Lungs, spleens, and brains were harvested from mice 4, 7, and 10 dpi (s.c., i.p., and i.n. routes) or 2, 4, and 6 dpi (i.c. route). Tissues were homogenized and renilla luciferase activity was measured as described in materials and methods. ANOVA and Tukey multiple comparison tests were used to calculate *P* values.

We and others have demonstrated that wild-type mice are not susceptible to infection by wild-type MuV strains [16–18, 27]. Tsurudome et al. demonstrated prolonged replication of a mouse-adapted variant of MuV in mice treated with anti-IFN antibody [17], suggesting that mice with defective IFN pathways might be more susceptible to MuV infection. IFN-α/βR^−/−^ mice have been used to study pathogenesis of viruses such as Junin virus, Dengue, and Ebola [19–21]. To determine the susceptibility of IFN-α/βR^−/−^ mice to MuV, mice were infected with MuV-IA containing a renilla luciferase reporter gene (rMuV-RLuc) via different infection routes. No significant RLuc activity was observed in tissues of IFN-α/βR^−/−^ mice infected subcutaneously (s.c.) with rMuV-RLuc compared to PBS mice (Fig 1A). IFN-α/βR^−/−^ mice infected via the intraperitoneal (i.p.) route had significantly enhanced RLuc activity in spleens 4 days post infection (dpi) (Fig 1B), however no virus was isolated from these tissue samples using plaque assay. IFN-α/βR^−/−^ mice infected via the intracranial (i.c.) route had ruffled fur, appeared sluggishness and lethargic, and were losing significant amounts of weight 4 dpi until reaching the human endpoint of 20% weight loss on 6 dpi (data not shown), when the experiment was terminated following the animal use protocol approved by our IACUC. In Fig 1C, increased luciferase activity was observed 6 dpi in brain and spleen tissues of infected mice. Virus titers of brain homogenates were 1.76x10^4^ PFU/mL and 1.65x10^4^ PFU/mL as determined using plaque assay, suggesting replication of rMuV-RLuc in these tissues. Plaques were not observed in the spleen homogenates. IFN-α/βR^−/−^ mice infected intranasally (i.n.) had significantly increased luciferase activity in lungs 4 dpi (Fig 1D), with a mean viral titer of 91 PFU/mL.

### MuV-IA titers in lungs of i.n. infected IFN-α/βR^−/−^ and wild-type C57BL/6 mice

**Fig 2:**
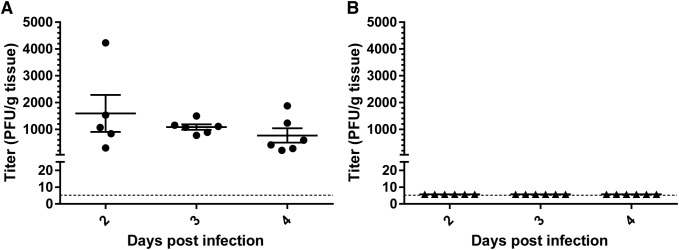
Lung viral titers of IFN-α/βR^−/−^ and C57BL/6 mice challenged with MuV-IA. (A) IFN-α/βR^−/−^ and (B) wild-type C57BL/6 mice were challenged i.n. with 1x10^6^ PFU MuV-IA in 100μL. Lungs were harvested on 2, 3, and 4 dpi. Tissues were weighed and homogenized and viral titers were determined by plaque assay in duplicate. The limit of detection of 5.8 PFU/g tissue is represented by the dashed line.

To determine lung viral loads in infected IFN-α/βR^−/−^ mice over time, mice were infected i.n. with 1x10^6^ PFU of MuV-IA in 100 μL and lung homogenates were collected at 2, 3, and 4 dpi. A peak mean viral titer of 1.6x10^3^ PFU/g lung tissue was observed at 2 dpi, followed by a non-significant decline until 4 dpi (Fig 2A). To confirm the absence of MuV-IA infection in wild-type C57BL/6 mice, viral titers were measured in C57BL/6 mice with MuV-IA as described above. No viral plaques were observed in lung homogenates collected at all time points, suggesting that viral titers were below the limit of detection (LOD) of 5.8 PFU/g tissue (Fig 2B).

### Passive transfer of serum from immunized mice reduces viral burden in IFN-α/βR^−/−^ mice

Previously, BALB/c mice have been used to generate antibody responses to MuV-IA following infections by the i.n. and intramuscular routes [16, 27]. To generate MuV-specific antibody, BALB/c mice were immunized i.n. with 1x10^6^ PFU of MuV-IA in a volume of 100 μL or mock immunized with 100 μL PBS. Mice were boosted at 3 and 6 weeks post immunization (wpi), then bled at 8 wpi. Serum was extracted and pooled for each group. A plaque reduction neutralization titer of 1:27 was measured for the pooled serum from the MuV-IA immunized mice.

**Fig 3:**
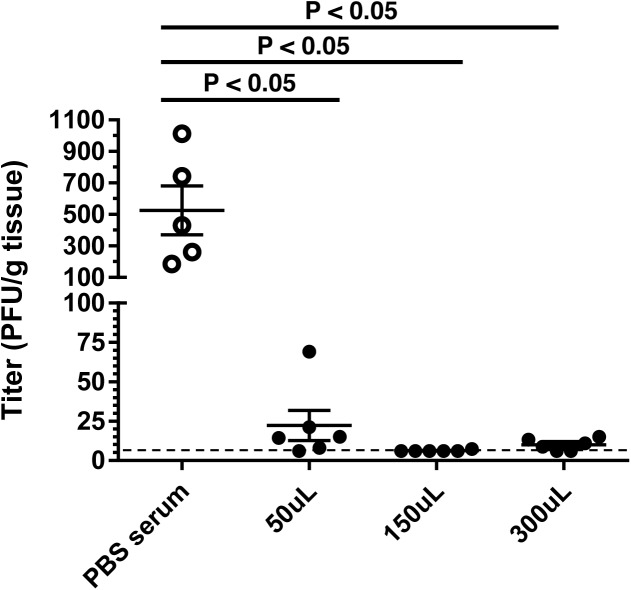
Reduced lung viral titers of challenged IFN-α/βR^−/−^ mice that received passive transfer of serum from MuV-IA-immunized mice. BALB/c mice were immunized i.n. with 1x10^6^ PFU of MuV in a volume of 100 μL or mock immunized with 100 μL PBS. Mice were boosted at 3 and 6 wpi, and serum was collected and pooled at 8 wpi. Naïve IFN-α/βR^−/−^ mice received passive transfer of 300 μL i.p. of inactived serum from MuV-IA immunized mice (50 μL and 150 μL groups received serum diluted in PBS for 300 μL total volume), or 300 μL i.p. inactivated serum from PBS immunized mice. Mice were challenged 24 hours post passive transfer i.n. with 1x10^6^ PFU of MuV-IA in a volume of 100 μL. Lungs were collected and homogenized 2 dpi and viral titers were determined by plaque assay in duplicate. The limit of detection of 5.9 PFU/g tissue is represented by the dashed line. ANOVA and Tukey multiple comparison tests were used to calculate *P* values.

To access the protective efficacy of the serum, various amounts of pooled serum from the immunized mice were passively transferred i.p. to naïve IFN-α/βR^−/−^ mice 24 hours prior to i.n. infection with MuV-IA. Viral burden in the lungs was measured 2 dpi. The viral burden in lungs from all mice that received serum from MuV-IA immunized mice was significantly reduced compared to mice that received serum from PBS immunized mice (Fig 3). Only one mouse of the 18 that received MuV-IA immunized serum had a viral titer greater than 25 PFU/g lung tissue and 8 had viral titers below the LOD. These results suggest that passive transfer of serum from MuV-IA immunized BALB/c mice can reduce the viral burden in IFN-α/βR^−/−^ mice infected i.n. with MuV-IA.

### Passive transfer of purified IgG from immunized mice reduces viral burden in IFN-α/βR^−/−^ mice

To determine the protective efficacy of IgG in our IFN-α/βR^−/−^ mouse model, BALB/c mice were immunized i.n. with 1x10^6^ PFU of MuV-IA in a volume of 100 μL to generate MuV-specific IgG antibody. Mice were boosted at 3 and 5 wpi and serum was pooled at 8 wpi. IgG was purified from the pooled serum and dialyzed in PBS. Serum eluate was also collected during IgG purification. A final concentration of 0.92 mg/ml with a plaque reduction neutralization titer of 1:32 was measured for the purified IgG antibody and a neutralization titer of 1:4 was measured for the serum eluate. To quantify MuV-specific antibody isotypes in the serum eluate and purified IgG samples, ELISAs were completed using plates coated with MuV-protein or antibody isotype standards (Table 1).

**Table 1:** Summary of antibody passive transfer experiment.

Antibody Isotypes	PBS	Serum Eluate	Purified IgG
**IgG (μg/mL)**	−	2.58	43.74
**IgA (μg/mL)**	−	2.24	< 0.01^a^
**IgM (μg/mL)**	−	0.54	0.01
**Mean lung titer per treatment group**	4631 PFU/g	614 PFU/g	12 PFU/g

^a^ Sample was below the limit of detection (LOD) of 0.01 μg/mL.

**Fig 4:**
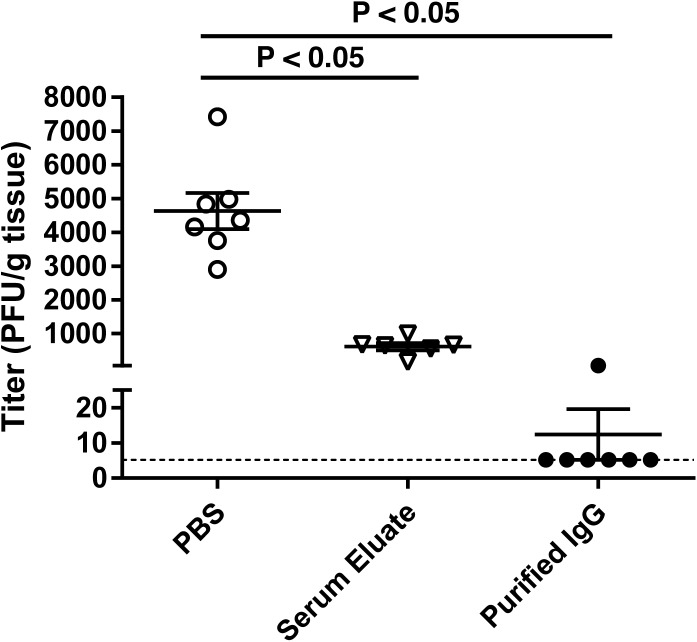
Reduced lung viral titers of challenged IFN-α/βR^−/−^ mice that received passive transfer of serum eluate and purified IgG from MuV-IA-immunized mice. BALB/c mice were immunized i.n. with 1x10^6^ PFU of MuV-IA in a volume of 100 μL. Mice were boosted at 3 and 5 wpi, and serum was collected and pooled at 8 wpi. IgG was purified by affinity chromatography as described in materials and methods and dialyzed in PBS. Column eluate (referred to as serum eluate) was collected during IgG purification. Naïve IFN-α/βR^−/−^ mice received passive transfer i.p. of 1 mL PBS, serum eluate, or purified IgG. Mice were challenged 24 hours post passive transfer i.n. with 1x10^6^ PFU of MuV-IA in a volume of 100 μL. Lungs were collected and homogenized 2 dpi and viral titers were determined by plaque assay in duplicate. The limit of detection of 5.4 PFU/g tissue is represented by the dashed line. ANOVA and Tukey multiple comparison tests were used to calculate *P* values.

Naïve IFN-α/βR^−/−^ mice received 1 mL of PBS, serum eluate, or purified IgG i.p. 24 hours prior to i.n. infection with MuV-IA. Viral burden in the lungs was measured 2 dpi. The viral burden in lungs from all mice that received purified IgG was significantly reduced compared to mice that received PBS (Fig 4). Of the 7 mice that received purified IgG, 6 had viral titers below the LOD in their lungs. Serum collected 2 dpi was used to measure plaque reduction neutralization titers. A range of titers from 1:2 to 1:32 was detected in mice that received serum eluate or purified IgG, and all mice with a plaque reduction neutralization titer ≥ 1:16 had no detectable virus in their lungs (Table 2).

**Table 2:** Neutralizing antibody titers in the antibody transferred mice before challenge.

Group	Mouse number	50% reduction titer by PRNT	Lung titer (PFU/g tissue)
**Serum Eluate**			
	42723–2	1:2	923
	42723–3	1:2	663
	42723–4	1:2	645
	42721–2	1:2	549
	42723–1	1:3.4	670
	42721–1	1:4	164
	**Mean ± SEM**	**1:2.6 ± 0.4**	**614 ± 109**
**Purified IgG**			
	42914–2	1:4	LOD^a^
	42914–4	1:4	LOD
	41606–2	1:8	56
	42914–1	1:16	LOD
	42914–3	1:16	LOD
	41606–1	1:32	LOD
	41606–3	1:32	LOD
	**Mean ± SEM**	**1:16 ± 4.5**	**12 ± 7**

^a^ Samples that were below the LOD of 5.4 PFU/g tissue were considered negative.

## Discussion

Recently, MuV outbreaks have occurred among highly vaccinated populations in the United States. A better understanding of the protective immune responses to MuV is needed to evaluate the efficacy of vaccines. The lack of a small animal model has been an impediment to our ability to understand the outbreaks in vaccinated populations as well as to develop new vaccine candidates.

In this study, we evaluated IFN-α/βR^−/−^ mice for their susceptibility to infection with MuV-IA. Transient replication of a mouse-adapted strain has been shown following infection by the natural route [17], however, wild-type MuV strains are not known to replicate well or cause illness in adult mice [16–18]. To the best of our knowledge, this is the first time that viral titers have been reported in tissues of mice infected with a clinical outbreak strain. Infectious virus was not detected in lung, spleen, or brain tissue of IFN-α/βR^−/−^ mice infected via intraperitoneal or subcutaneous routes. In mice infected by the intracranial route, virus was detected in brains on 6 dpi at a titer several logs higher than that of input virus, indicating that the virus replicates in this tissue. Even though virus was not detected in the spleens of mice injected with virus intracranially, renilla luciferase activity was considerably greater in these tissues than in that of the mock-infected mice (Fig 1C). This data suggests that intracranial infection with MuV-IA might somehow allow the trafficking of immune cells from the infected brain to the spleen. One advantage of using a recombinant MuV expressing luciferase is that expression of luciferase indicates potential of viral transcription activity. MuV is a neurotropic pathogen (reviewed in reference [28]). The use of the intracranial infection route may serve as a model to study MuV pathogenesis in the neurological system. The model can possibly be used as a system to evaluate potential small molecule anti-viral drug candidates. However, due to the brain blood barrier, the model may be of limited use to evaluate efficacy of vaccine candidates.

IFN-α/βR^−/−^ mice infected by the intranasal route resulted in peak viral titers in the lungs at 2 dpi. Infectious virus was not detected in lungs of infected control C57BL/6 mice, suggesting that innate immunity plays a protective role in mice with functional interferon responses. While intranasal infection of IFN-α/βR^−/−^ mice may be used as a model to study clinical MuV strains *in vivo*, careful use of the model and interpretation of results is needed due to the important role of IFN in viral pathogenesis.

One challenge for developing a MuV vaccine is that correlates of protective anti-MuV responses have not been determined. Virus-specific serum IgM, IgA, and IgG are all detectable following natural MuV infection in humans. Serum IgM and IgA levels have been shown to decline to undetectable levels by 8 weeks following symptom onset, however serum IgG levels can still be detected years later [29–31]. A mumps neutralizing antibody titer predictive of protection against infection or disease has not been established. A better understanding of protective responses to mumps is important for development of improved vaccines. In this study, we used our intranasal IFN-α/βR^−/−^ mouse model to evaluate the protective efficacy of antibodies against mumps infection. Passive transfer of sera from MuV-infected mice provided protection in naïve mice. Further purification of sera from immunized mice, which contained MuV-specific IgG, IgA, and some IgM in the serum, protected mice against MuV infection, indicating that antibody can be protective. However, we were unable to determine a numerical value for antibody titers needed for complete protection: all mice with a titer of 1:32 were completely protected while a mouse with antibody at 1:8 was not completely protected. Passive transfer of serum eluate resulted in a significant reduction in lung viral titers compared to control mice, likely because it also contained MuV-specific IgG, IgA and IgM. Secretory IgA with neutralizing activity has been shown to be produced in the nasal cavity of humans following natural mumps infection and vaccination [32, 33]. Elucidation of the protective role of each antibody isotype may benefit future vaccine development. Our work has not addressed the role of cellular immune responses in protection. Furthermore, it is not known whether our data from mice can be applied in humans.
